# Writing Beyond the Academic Context: Exploring Writing Among Public Health Practitioners

**DOI:** 10.1177/00333549251341238

**Published:** 2025-07-15

**Authors:** Robert Larsson, Jennifer Beard, Maria Norfjord van Zyl

**Affiliations:** 1 Division of Public Health Sciences, School of Health, Care and Social Welfare, Mälardalen University, Västerås, Sweden; 2 Department of Global Health, Boston University School of Public Health, Boston, MA, USA

**Keywords:** audience, communication, professional, public health, writing

## Abstract

**Objectives:**

Public health work involves diverse types of writing to communicate health messages to various audiences. Clear, concise, audience-oriented writing is essential, yet public health practitioners often receive little training in effective writing. This study explores the writing public health practitioners do in their everyday work.

**Methods:**

We emailed a web-based questionnaire in April 2024 to a sample of public health practitioners working in municipalities, regional health departments, governmental agencies, and nongovernmental organizations in 5 regions in Sweden. The questionnaire asked about writing, support, professional development, and writing self-efficacy. We analyzed data using descriptive statistics and content analysis.

**Results:**

Seventy-two public health practitioners responded to our questionnaire. The most common types of writing that respondents reported engaging in were presentations (88.9%), reports (59.7%), and decision-making documents (47.2%). Scientific articles (75.0%), blog posts (69.4%), and opinion pieces (56.9%) were the least common. The most common target audiences were politicians, followed by managers and citizens. Colleagues provided the most common source of writing support, followed by communication officers and managers. Most practitioners reported a desire to develop their professional writing skills and achieve high self-efficacy in public health writing.

**Conclusions:**

Public health practitioners in Sweden write more for politicians and managers than for the public. Expanding practitioners’ writing skills beyond operational and technical document writing to include public-facing writing will benefit the public health profession by opening communication channels to diverse audiences. Communicating more clearly with public audiences can improve health literacy, promote health for all, and strengthen the effectiveness of public health initiatives.

Public health work involves diverse writing tasks to communicate health-oriented messages to multiple audiences.^1^ Yet, public health practitioners do not always receive the training needed to become effective writers and communicators. The same applies to students and university professors who are used to academic writing but rarely have formal training in communicating public health concepts and evidence in writing. Moreover, professors are not always prepared to teach academic writing, much less public health writing.^2-5^ This lack of training might be somewhat surprising because communication is a core competency in public health promoted by the Association of Schools of Public Health in the European Region^6^ and the US Council on Education for Public Health (CEPH).^7,8^

Communication competency refers to both written and oral communication. However, the European List of Core Competencies mainly emphasizes the ability to use written media, audiovisual techniques, and social media to communicate public health messages to lay, professional, academic, and political audiences.^6^ The CEPH competencies have similar limitations in that they assume that students already understand and have practiced writing a wide array of public health documents for many different audiences. That students have practiced a wide array of writing is often a faulty assumption.

Clear, concise, purpose-driven, and audience-focused writing is the foundation of all forms of health communication. Writing is the structured expression of thoughts, ideas, and information using symbols, typically words. Every time we sit down to write, we set out to construct something from nothing. We type words across our screen, building sentences, paragraphs, and documents to convey information, stories, and analysis.^9^

We define public health writing as writing that aims to maintain and improve the health of groups and populations. Public health writers aim to educate and create change by informing, influencing, and motivating action in various audiences.^4^ The history of public health is full of more or less effective examples of public health writing. We have only to revisit key moments during the COVID-19 pandemic when public health leaders and agencies in Sweden, the United States, and around the world struggled to communicate the emerging science in a way that the public could easily understand and trust.^10^ These examples included overly complex or ambiguous messages about how best to prevent infection. The communication challenges and failures of the COVID-19 pandemic gave urgency to a quiet movement focused on training public health students to be better writers, which has been growing for some years in academic programs that train future public health practitioners.^7,11-13^

Public health is political, and this notion is something all public health practitioners need to keep in mind when communicating with the public (in whatever format that communication takes place). The COVID-19 pandemic demonstrated just how political public health can become, and clear, precise, audience-oriented writing is an antidote to confusion and misinformation.^14^

Given this background, public health educators need to equip future practitioners with professional writing as a key skill. Since 2014, the de Beaumont Foundation has been conducting the Public Health Workforce Interests and Needs Survey (PH WINS) to document training strengths and gaps.^15,16^ The survey has found that public health communication skills are consistently among the top 10 training needs in the US public health workforce. Sweden has no equivalent to PH WINS. As a result, little is known about the public health workforce in Sweden, including knowledge gaps in writing skills. We explored the writing public health practitioners do in their everyday work. To our knowledge, it is the first study in Europe to examine public health writing among practitioners.

## Methods

### Design, Sample, and Data Collection

This study used a cross-sectional design to explore public health writing among practitioners in 5 regions in central Sweden. Following a stratified purposeful sampling approach, we considered the regions as strata to capture data on differences among organizations, with each stratum constituting a fairly homogeneous sample.^17^ In April 2024, we emailed a web-based questionnaire, along with information on the aim, research ethics, and practical value of the study, to public health practitioners working in municipalities, regional health departments, governmental agencies (ie, county administrative boards), and nongovernmental organizations (NGOs) in each region. We used professional email addresses from each organization’s website. In total, we sent 154 email invitations to practitioners providing public health services in the 5 regions. We collected data in April and May 2024.

### Questionnaire

The questionnaire included 19 open- and closed-ended questions and was in Swedish (English translation of the questionnaire is provided in Supplemental Material). The questions collected information on participant demographic characteristics and job details, such as position and organization. The questions also focused on types of documents respondents most commonly wrote, audiences, and time spent writing on the job. Other questions explored available writing support, writing skills development, and barriers to writing. Finally, we assessed writing self-efficacy by a single statement on confidence in writing ability in documents relevant to public health work, with responses ranging from 1 (strongly disagree) to 5 (strongly agree) on a 5-point Likert scale. Before distributing the email, we and a fellow public health educator and researcher reviewed the questionnaire for face validity, clarity, and relevance.

### Data Analysis

We analyzed data by using descriptive statistics and calculating the frequencies, percentages, means, and SDs of closed-ended questions. We used summative content analysis to analyze open-ended responses, involving counting, comparisons, and keyword interpretations.^18^ One author (R.L.) first analyzed all responses, and another author (M.NvZ.) reviewed the responses. Discrepancies were resolved through discussion. We summarized the responses in descriptive text and reviewed the final descriptions of the open-ended responses.

### Ethical Considerations

We followed accepted research ethics principles, informing participants about the study’s aim and design, and we obtained informed consent in the survey system before participants could begin answering questions. Participants were also informed about confidentiality, data handling, and the right to withdraw from the study at any time. As a professional survey without sensitive personal information, the study did not need approval under the Swedish Act (2003:460) concerning ethical review of research involving humans.^19^

## Results

Seventy-two public health practitioners (46.8% of those invited) responded to the questionnaire (Table 1). Most respondents were female (n = 62; 86.1%), and the mean (SD) age was 44.7 (9.0) years. The most common type and level of higher education was a master’s degree or doctorate in public health (n = 27; 37.5%). Practitioners had a mean (SD) of 6.5 (7.2) years of work experience in their current position. The most common job position was development leader (n = 17; 23.6%), followed by public health strategist (n = 10; 13.9%) and health counselor/consultant (n = 8; 11.1%) (eTable 1 in Supplemental Material). Primary employers were municipalities (n = 27; 37.5%), regional health departments (n = 19; 26.4%), and NGOs (n = 18; 25.0%).

**Table 1.** Characteristics of public health practitioner respondents (N = 72) to a questionnaire on public health writing, Sweden, April–May 2024

Characteristic	No. (%) of respondents
Sex (n = 72)	
Female	62 (86.1)
Male	8 (11.1)
No response	2 (2.8)
Age group, y (n = 71)	
20-29	2 (2.8)
30-39	19 (26.8)
40-49	29 (40.8)
50-59	17 (23.9)
≥60	4 (5.6)
Education (n = 72)	
Bachelor of public health degree	21 (29.2)
Master of public health or higher degree	27 (37.5)
University degree in social sciences	8 (11.1)
University degree in other subjects	13 (18.1)
Other educational background	3 (4.2)
Current job position (n = 72)	
Development leader	17 (23.6)
Public health strategist	10 (13.9)
Sustainability strategist	4 (5.6)
Project leader/process leader	3 (4.2)
Health counselor/consultant	8 (11.1)
Sports consultant	5 (6.9)
Other (eg, strategist, coordinator, analyst, expert, business developer, manager)	25 (34.7)
No. of years in current job position (n = 71)	
0-5	46 (64.8)
6-10	10 (14.1)
11-15	5 (7.0)
≥16	10 (14.1)
Organization (n = 72)	
Governmental organization	8 (11.1)
Municipality	27 (37.5)
Nongovernmental organization	18 (25.0)
Regional health department	19 (26.4)

### Types of Public Health Writing, Audiences, and Time Spent Writing

The 5 most common types of public health writing reported were PowerPoint presentations (n = 64; 88.9%), reports (n = 43; 59.7%), decision-making documents (n = 34; 47.2%), project plans (n = 34; 47.2%), and policy documents (n = 32; 44.4%) (Table 2). Respondents indicated that the least common types of writing they do in their jobs were scientific articles for peer-reviewed journals (n = 54; 75.0%), blog posts (n = 50; 69.4%), opinion pieces (n = 41; 56.9%), abstracts (n = 38; 52.8%), and popular science abstracts/articles (n = 30; 41.7%).

**Table 2.** Types of public health writing of public health practitioner respondents (N = 72) to a questionnaire, Sweden, April–May 2024

Type of document^a^	Common type of writing, % (rank)	Most common type of writing, %	Least common type of writing, %
PowerPoint presentation	95.8 (1)	88.9	0
Report	76.4 (2)	59.7	6.9
Decision-making document^b^	70.8 (3)	47.2	5.6
Project plan^b^	69.4 (4)	47.2	4.2
Policy document	68.1 (5)	44.4	11.1
Web text	65.3 (6)	30.6	4.2
Newsletter^b^	50.0 (7)	26.4	5.6
Fact sheet	43.1 (8)	19.4	5.6
Social media (eg, Facebook, Instagram, LinkedIn, X)	43.1 (8)	15.3	22.2
Brochure	41.7 (10)	23.6	12.5
Press release	34.7 (11)	4.2	20.8
Literature review	22.2 (12)	9.7	23.6
Poster	18.1 (13)	11.1	19.4
Popular science abstract/article^b^	8.3 (14)	2.8	41.7
Policy brief	6.9 (15)	4.2	29.2
Opinion article	5.6 (16)	2.8	56.9
Abstract	1.4 (17)	0	52.8
Blog post	1.4 (17)	0	69.4
Scientific article	0 (19)	0	75.0

a Of 19 document types described in an article by Mackenzie,^1^ respondents were asked about 11 document types in our questionnaire. In the adapted question on types of documents, 4 other document types were included along with other types of written communication.

b Document types added to the questionnaire.

Politicians were the most common audience (n = 47; 65.3%) followed by managers (n = 46; 63.9%) and citizens (n = 41; 56.9%). Respondents also reported writing for colleagues (n = 39; 54.2%) and target groups such as children, adolescents, or parents (n = 29; 40.3%). Most respondents (n = 32; 45.7%) reported spending 1 or 2 working days (20%-40% of their working time) writing in an average week. Just 6% reported spending >3 working days writing per week (ie, >60% of their working time) (Table 3).

**Table 3.** Writing time, writing skills, and writing self-efficacy of public health practitioner respondents (N = 72) to a questionnaire on public health writing, Sweden, April–May 2024

Variable	No. (%)	Writing skills and self-efficacy scales, mean (SD)^a^
Writing time^b^ of total working time, % (n = 70)		
<20	20 (28.6)	—
20-40	32 (45.7)	
41-60	14 (20.0)	
61-80	4 (5.7)	
>80	0	
Use of writing knowledge and skills on the job (n = 72)		
To a very small degree	2 (2.8)	4.1 (0.9)
To a small degree	1 (1.4)	
Partly	11 (15.3)	
To a high degree	34 (47.2)	
To a very high degree	24 (33.3)	
Work opportunities for writing skill development (n = 72)		
To a very small degree	7 (9.7)	2.8 (1.0)
To a small degree	17 (23.6)	
Partly	34 (47.2)	
To a high degree	9 (12.5)	
To a very high degree	5 (6.9)	
Writing self-efficacy (n = 72)		
Strongly disagree	0	4.0 (0.8)
Agree to a low degree	2 (2.8)	
Partially agree	15 (20.8)	
Agree to a high degree	38 (52.8)	
Strongly agree	17 (23.6)	

Abbreviation: —, does not apply.

a The 2 writing skill variables were measured on a 5-point Likert-type scale, from 1 (to a very small degree) to 5 (to a very high degree), with higher scores indicating more use and development opportunities. Writing self-efficacy was assessed by a single statement on confidence in writing ability in documents relevant to public health work, also rated on a 5-point Likert-type scale, with higher scores indicating greater self-efficacy.

b Writing time is translated to working days in the text.

### Support, Professional Development, and Writing Self-Efficacy

Colleagues were the most common source of on-the-job writing support (n = 67; 93.1%), followed by communication officers (n = 50; 69.4%) and immediate managers (n = 45; 62.5%). Nearly one-fourth of respondents (n = 16; 22.2%) reported using artificial intelligence (AI) writing tools to support their writing, and AI was most used by practitioners in NGOs and regional health departments (n = 13; 81.3%).

With regard to professional development, most public health practitioners reported that they build their writing knowledge and skills to a high or very high degree (n = 58; 80.6%) on the job (Table 3). Nearly half (n = 34; 47.2%) said their job partly offers opportunities to develop writing skills, whereas one-third (n = 24; 33.3%) reported such opportunities as low or very low. Moreover, 56.5% (n = 39) said that they needed to develop their writing skills, compared with 43.5% (n = 30) who reported no such need. Most public health practitioners (n = 55; 76.4%) reported high writing self-efficacy in their professional roles (mean [SD] = 4.0 [0.8]).

### Open-Ended Responses

Public health practitioners noted that they developed their writing skills through a combination of academic training and work experience, with academia laying the foundations and work shaping practical and targeted writing. Practitioners described writing as a key job skill, with more experienced colleagues helping younger colleagues to express themselves concisely and effectively. One practitioner expressed this difference between academic and professional writing: “You received grounding in writing at university, but at work, texts should be much shorter and rarely contain references.”

The main barriers to effective writing were lack of time, limited communication resources, and the challenges of adapting public health messages to various audiences. One practitioner expressed the following barriers: “Public health sciences contain concepts that are not generally accepted, which makes it difficult to interact with other colleagues who do not have the same expertise. Sometimes, it is also difficult to adapt texts to different target groups, especially politicians.”

Practitioners explained that their job offers opportunities to develop writing skills through in-house training and communication support, but most said that development mainly takes place through practical work and collegial feedback. Practitioners also aspired to develop understandable and educational writing styles for different audiences, especially concerning explaining complex topics and scientific uncertainties. Finally, one practitioner noted a personal writing goal was to “formulate messages for different audiences and express myself pedagogically without always having to use jargon.”

## Discussion

The main results of our study were that public health practitioners usually write presentations, reports, project plans, and decision and policy documents, with politicians and managers as their primary audience. This writing reflects the creation of operational and technical documents to facilitate decision-making and report on the design, implementation, and evaluation of public health projects, interventions, and policies. The audiences for this type of writing are most often managers, politicians, and funders.

Practitioners also reported writing for citizens in the form of fact sheets, brochures, and other educational documents. But public engagement did not typically extend to writing public texts, such as opinion pieces and blog posts. It was also unusual for Swedish public health practitioners to write for the scientific community (eg, scientific articles). Thus, a challenge for practitioners, depending on their job descriptions, might be to find a balance between the demand for operational and technical document writing and more public-facing writing with different communities or the public as the primary audience.

The practitioners reported writing frequently for politicians and mostly writing decision and policy documents, where the latter refers to official organizational policies, such as municipal public health policies. However, they wrote few policy analyses and briefs (ie, analyses of existing policies and research-based recommendations to policy makers), which are common in public policy writing.^20^ This lack of demand may be explained by organizational culture, where policy analysis and briefs are more common in national government (ie, federal level) and scientific professional roles than in municipalities and regional health departments.

Public health practitioners also have expertise to share with the scientific community. To some, writing peer-reviewed articles may feel daunting and beyond their skill level. Nevertheless, contributing to the evidence base and to scientific conversations can help close the gap between research and practice.^21^ Such writing can also be done collaboratively and with the support of researchers. Similarly, practitioners and researchers should share their work and knowledge with general readers who do not have scientific or public health expertise. This type of writing is different and will entail different types of writing training.

We recommend broadening the public health practitioner’s ability to write multiple types of documents^1^ for expert, political, and lay audiences.^6^ This broader writing also includes writing on social media platforms to correct misinformation and add balance and nuance to heated and evidence-free public health debates.^22^

Our results show that public health practitioners primarily receive support for their writing from colleagues and communication officers. One-quarter of respondents also turned to AI tools for assistance. A recent study among public health students and instructors showed that the thoughtful use of AI in the classroom can help prepare students for the future as professionals, but the results also illustrate the risks of inaccurate information and unethical behavior.^23^ For example, AI may save time on administrative tasks, allowing more time to be spent designing evidence-based and effective public health messaging; there is, however, a need to study how AI tools can be used to support public health writing in the future.

Our results also show that many public health practitioners are interested in developing their writing skills, and most report high levels of writing self-efficacy. A way forward for professional development is to create writing groups in which public health practitioners can work collectively to build their writing experience and skills. Practice-based knowledge shows that engaging in writing groups can help writers learn new skills, share frustrations and successes, reduce anxiety, and increase productivity.^24^

Building the writing skills of public health practitioners is critical to professional development, particularly in today’s politicized and polarized social media culture.^25,26^ To earn audience trust, public health practitioners need to be able to tailor messages that speak to their priorities in language that is engaging and easy to understand. For example, public health practitioners can use stories to bring to life and make the connection between personal health and public health.^27^ Such connections make it easy for readers to see how public health topics and evidence are relevant to their lives and aspirations.

### Limitations

Our study had several limitations. First, it was limited by the focus on a single country and the small sample size. Second, the distribution of the questionnaire may have limited participation from public health practitioners who do not consider themselves to be writers even though they write in their jobs. Those most interested in writing and communication may have been most likely to complete the questionnaire, potentially resulting in more positive responses and social desirability bias.

Third, because of the lack of existing questionnaires about public health writing, we developed and piloted the questions in our study. Although our questionnaire was based on previous literature, nonvalidated questions were a study limitation. Fourth, when reviewing the data, we also noticed that we did not ask about some common types of writing (eg, protocols). Fifth, because our primary interest was public health writing, we chose not to include email among the document types, which might be considered another limitation of the study. Despite these limitations, our study captured novel information that can be used in Sweden and elsewhere to advocate for and design writing support for public health practitioners.

### Implications for Education and Practice

Our study has several implications for education and practice. Previous literature emphasizes the need to strengthen communication skills within the future public health workforce.^25^ Public health schools and programs in Sweden and elsewhere can better prepare their students for the workforce by building writing training explicitly into the curriculum and course syllabi. Practical writing assignments can give students experience with writing professional documents for specific audiences.^12^ August and Anderson^13^ created a useful framework for designing practical public health writing assignments.

Our results show that most public health practitioners want to develop their writing skills. Writing training and support can be offered through university continuing education, certificate programs, and massive open online courses (eg, Coursera, edX) focused on health writing, ad hoc webinars, planned workshops, and workplace writing groups.^24,28^ Such professional development initiatives offer valuable opportunities for public health practitioners and academics to collaborate, share expertise, build confidence, and encourage approaching writing as a lifelong practice.

### Future Research

We suggest future research along four lines. First, the professional writing of public health practitioners can be examined in different countries and contexts. Because public health work is organized and conducted differently in various cultural contexts, the way public health writing is conducted might also differ. For example, interaction and knowledge exchange with multiple audiences can vary. Exploring public health writing in different cultural contexts allows us to increase our knowledge of the various conditions under which public health communication takes place. Second, public health writing can take on the perspectives of different employers and can include employer expectations about the writing and communication skills that will be most useful for the future public health workforce. This understanding helps enhance the communication skills of public health practitioners, especially in a world that uses diverse methods of public health messaging.

Third, attitudes and use of AI tools for effective public health writing can be investigated in the public health workforce. Finally, public health writing can be explored from the perspective of target audiences, community members, and populations, as they are the final recipients of much of the writing done by public health practitioners. This research is crucial as we can better target our writing to educate and create health-related actions among various audiences.

## Conclusions

Public health practitioners in Sweden tend to write more for politicians and managers than for the public. Practitioners need to broaden their writing skills beyond operational and technical document writing to include targeted and public-facing writing for diverse communities and populations. This broader writing implies expanding the type of documents that public health practitioners write. One advantage is that practitioners report high self-efficacy in writing documents relevant to public health work. Developing a wide range of writing skills is beneficial in meeting varying levels of health literacy, promoting health for all, and making a public health impact.

## Supplemental Material

sj-docx-1-phr-10.1177_00333549251341238 – Supplemental material for Writing Beyond the Academic Context: Exploring Writing Among Public Health Practitioners Supplemental material, sj-docx-1-phr-10.1177_00333549251341238 for Writing Beyond the Academic Context: Exploring Writing Among Public Health Practitioners by Robert Larsson, Jennifer Beard and Maria Norfjord van Zyl in Public Health Reports®
