# Supplementary material for: Writing Beyond the Academic Context: Exploring Writing Among Public Health Practitioners
Source: Public Health Rep. 2025 Jul 15;140(4):412–8. doi: 10.1177/00333549251341238 (PMC12264250; doi:10.1177/00333549251341238)
Supplement: sj-docx-1-phr-10.1177_00333549251341238 – Supplemental material for Writing Beyond the Academic Context: Exploring Writing Among Public Health Practitioners [file sj-docx-1-phr-10.1177_00333549251341238.docx]

**Supplemental material**

**Table 1. Descriptions of job positions in the Swedish public health system.**

| **Job position** | **Job description** |
| --- | --- |
| Development leader | Development leaders are usually based in the regional government, and the job involves a high degree of cross-sectoral work. Their role is primarily strategic and may involve designing policies, programs, and interventions. People working in this role typically oversee needs assessment, project planning, implementation, and evaluation. |
| Public health strategist | Public health strategists work for municipal governments. Their work is similar to that of regional development leaders with whom they can collaborate. They engage in a high degree of cross-sectoral work.  Depending on the municipality where they are based, these employees may hold the title of public health strategist, public health coordinator, or public health developer. The differences in the titles are driven more by municipality tradition than by differences in the scope of work. |
| Sustainability strategist | Sustainability strategists plan, implement, and evaluate activities related to the United Nations Agenda 2030 framework and the 17 global Sustainable Development Goals. They are based in municipal or regional government or county administrative boards. They collaborate across multiple sectors to guide the ecological, economic, and social sustainability dimensions of improving health and wellbeing. |
| Health counselor/consultant | Health counselors/consultants are based at specific wellness centers. Their role is to guide, inform, and motivate people who face varying health choices. The work focuses on supporting individual healthy behavior related to physical activity, healthy foods, alcohol and tobacco. The support they provide is guided by national guidelines for disease prevention methods. |
| Sports consultant | Sports consultants inform, guide, and support sports clubs and organizations. Lately, the work has broadened to areas like public health, gender equality and inclusion. The job is both strategic and operational and may involve designing policies, programs and various projects. Sports consultants are based in civil society organizations and work under the Swedish Sports Confederation. |

**The Questionnaire**

Introductory questions

**Q1. What is your current job position (job title)?**

*Response options:*

- Development leader
- Public health coordinator
- Public health developer
- Public health planner
- Public health strategist
- Sustainability strategist
- Prevention coordinator
- Project manager/process manager
- Health counselor/consultant
- Sports consultant
- Other, what? __________________

**Q2. How many years have you worked in your current position (professional role)?**

*Response options:* Number of years? ______

**Q3. In which type of organization do you work?**

*Response options:*

- Municipality
- Regional government
- Non-governmental organization (association, volunteer organization, or similar)
- Private company
- Other (describe) ______________

Questions about writing

**Q4. What type of documents do you generally write in your professional role?** Select all possible types of documents.

*Response options:*

- Abstract
- Blog
- Brochure
- Decision-making documents
- Fact sheet
- Literature review
- Newsletter
- Opinion article
- Policy brief (summary of scientific knowledge and recommendations)
- Policy document
- Popular science abstract/article
- Poster
- PowerPoint
- Press release
- Project plan
- Report
- Scientific article for a peer-reviewed journal
- Social media (e.g., Facebook, Instagram, LinkedIn, X)
- Web text
- Other (describe) __________

**Q5. What type of documents do you primarily write in your professional role?** Select the five most common document types.

*Response options:*

- Abstract
- Blog
- Brochure
- Decision-making documents
- Fact sheet
- Literature review
- Newsletter
- Opinion article
- Policy brief (summary of scientific knowledge and recommendations)
- Policy document
- Popular science abstract/article
- Poster
- PowerPoint
- Press release
- Project plan
- Report
- Scientific article for a peer-reviewed journal
- Social media (e.g., Facebook, Instagram, LinkedIn, X)
- Web text
- Other (describe) __________

**Q6. What type of documents are you least likely to write in your professional role?** Select the five least common document types.

*Response options:*

- Abstract
- Blog
- Brochure
- Decision-making documents
- Fact sheet
- Literature review
- Newsletter
- Opinion article
- Policy brief (summary of scientific knowledge and recommendations)
- Policy document
- Popular science abstract/article
- Poster
- PowerPoint
- Press release
- Project plan
- Report
- Scientific article for peer-reviewed journal
- Social media (e.g., Facebook, Instagram, LinkedIn, X)
- Web text
- Other (describe) __________

**Q7. Which audiences do you primarily write for?** Please specify the three most common audiences.

*Response options:*

- Politicians
- Managers
- Colleagues
- Citizens (e.g., community members, municipality or regional inhabitants)
- Specific target groups (e.g., children/youth, parents etc.)
- Other target groups? __________

**Q8. Where have you learned to write the type of documents you write in your work?**

*Response option:* Open-ended response.

**Q9. What proportion (%) of your time is spent writing during an average working week?** (excluding e-mail)

*Response options:*

- Less than 20%
- 20-40%
- 41-60%
- 61-80%
- Over 80%

**Q10. I have high confidence in my ability to write documents that are relevant to my public health work.**

*Response options* (5-point Likert scale):

1. Strongly disagree
2. Agree to a low degree
3. Partially agree
4. Agree to a high degree
5. Strongly agree

Questions on support and development

**Q11. What support do you have access to when working on a writing project?**

*Response options:*

- Colleagues
- Team leader/project manager
- Immediate manager
- Communication officer/communication support
- AI tools
- Other technical support (describe) ________________
- Other support (describe) ________________

**Q12. Is there anything that hinders you in your writing?**

*Response option:* Open-ended response.

**Q13. Are you able to use your writing knowledge and skills in your work?**

*Response options* (5-point Likert scale):

1. To a very small degree
2. To a small degree
3. Partly
4. To a high degree
5. To a very high degree

***Follow-up question:*** Please describe how you use your writing knowledge and skills?

*Response option:* Open-ended response.

**Q14. Does your job offer opportunities to develop your writing skills?**

*Response options* (5-point Likert scale):

1. To a very small degree
2. To a small degree
3. Partly
4. To a high degree
5. To a very high degree

***Follow-up question:*** Can you describe in what way your employer offers opportunities to develop writing skills?

*Response option:* Open-ended response.

**Q15. Do you feel that you need to develop your writing skills?**

*Response options:*

- Yes
- No
- If yes, what type of support would be most useful to you? __________

Background questions:

**Q16. What is your age?**

*Response option:* Enter your age _______

**Q17. What is your gender?**

*Response options:*

- Female
- Male
- Non-binary
- Do not want to specify

**Q18. What is your highest level of education?**

*Response options:*

- Theoretical upper secondary education
- Practical upper secondary education
- Adult education
- Vocational college
- University education
- Public health sciences, bachelor level
- Public health sciences, one-year master’s/two-year master’s/PhD level
- Social sciences
- Other areas of university study
- Non-degree university courses
- Other education

If possible, please indicate the subject area/specialization of your education.

**Q19. Other comments**

*Response option:* Open-ended response.
